# Welcome to *Nursing Open*

**DOI:** 10.1002/nop2.1

**Published:** 2014-03-05

**Authors:** Roger Watson

I am pleased to announce the launch of *Nursing Open*, a peer‐reviewed, international, fully open access journal that welcomes articles on all aspects of nursing and midwifery practice, research, education and policy.

You have probably lost track of the number of open access nursing journals but you cannot have failed to notice the daily onslaught of invitations to submit to, edit or join the editorial board of a new open access publication. I often receive emails from flattered colleagues telling me they have received such an invitation and asking how they should respond. When I point out that I also received the same invitation, and that most of their colleagues likewise received it, they feel less flattered and usually take it no further. I would not blame you, therefore, if you questioned the need for another online open access nursing journal. What does *Nursing Open* offer, and how will it differ from other open access journals?

*Nursing Open* will be a journal that demonstrates quality, integrity and speed, and we expect to hear from authors and readers if we stray from these watchwords. We will strive to ensure that *Nursing Open* maintains the same high standards as other Wiley journals and stands out among online open access journals. We aim, for example, to be indexed by Thomson Reuters and achieve an impact factor within as short a time as possible.

In terms of our three watchwords, our goals are to achieve:

*Quality* – in launching *Nursing Open*, we have assembled an excellent team of associate editors and editorial board members. Each member of the team was carefully selected and has ‘signed up’ to the aims and scope of the journal. With few exceptions, the highest quality publications are not at the moment purely online or fully open access; we intend to publish a journal that is both of these, and is as good as any in the field. The journal's mission is to publish articles that contribute to the art and science of nursing and which have a positive impact on health, either locally, nationally, regionally or globally. As such, we will publish articles with scientific credibility and rigour, and coherence and clarity in the writing. Confirmatory and replication studies will be considered; however, the article must present new findings, which could include the reporting of negative findings.

*Integrity* – *Nursing Open* will operate according to the highest standards of authorship, peer review, editing and publishing. The ‘pay to publish’ aspect of the journal will be entirely separate from judging the scientific worth of articles. We provide a clear standard template for submission to which all articles must conform, and all submissions will be peer reviewed and edited. The process of publication and any problems or disputes arising throughout that process and after publication will be handled with fairness and equity according to the COPE guidelines.

*Speed* – we aim to provide a rapid route from submission to publication in *Nursing Open*: 5 weeks from submission to first decision and 5 weeks from acceptance to online publication. This will be achieved by a fair distribution of work to associate editors and intimate involvement of the editorial board in the peer reviewing process. As the journal grows, the size of the reviewing panel will increase accordingly to ensure that submissions are processed as quickly as possible. As soon as articles are accepted and produced, they will appear online with a digital object identifier and be immediately available for sharing with peers and colleagues.

The benefits of the publication model that *Nursing Open* is adopting are also worth highlighting. The scientific community as a whole invests a great deal of time and energy in publication, and for this investment to achieve the maximum impact, articles need to be disseminated as widely as possible. We realize that publication quality and free access at the point of use come at a price. Therefore, the pay to publish model operated by *Nursing Open*, offering the ‘gold route’ to open access, is one we believe offers the most rapid and sustainable way to share publications that meet the requirements of funding bodies and the scientific community. All articles published by *Nursing Open* are fully open access: freely available to read, download and share on publication. Articles are published under the terms of the Creative Commons Attribution License and copyright is retained by the author(s).

A further benefit of *Nursing Open*'s model is the more efficient use of the peer review process. *Nursing Open* is supported by other journals published by Wiley, and these journals will give authors whose articles are not accepted the opportunity to transfer their article (and the peer reviews if applicable) to *Nursing Open*. If peer reviews are available, the *Nursing Open* editorial team will assess them and decide whether or not to seek further peer reviews. This eases the burden on reviewers while ensuring a rigorous peer review system.

Therefore, welcome to *Nursing Open*, which we hope you will find a useful outlet for your research and a useful source of information, evidence and debate. Our Aims and Scope and Guidelines for Authors are available to view and you can meet the editorial team on the journal website at www.nursingopenjournal.com. Also, we hope that you will follow us on Twitter and read and contribute to the *Nursing Open* blog.

